# A circular network of purine metabolism as coregulators of dilated cardiomyopathy

**DOI:** 10.1186/s12967-022-03739-3

**Published:** 2022-11-18

**Authors:** Ge Wang, Rongjun Zou, Libao Liu, Zongtao Wang, Zengxiao Zou, Songtao Tan, Wenliu Xu, Xiaoping Fan

**Affiliations:** 1 Department of Cardiovascular Surgery, Guangdong Provincial Hospital of Chinese Medicine, the Second Affiliated Hospital of Guangzhou University of Chinese Medicine, Guangzhou, 510120 Guangdong China; 2 The Second Clinical College of Guangzhou, University of Chinese Medicine, Guangzhou, 510405 Guangdong China; 3 Department of Cardiothoracic Surgery, Third Affiliated Hospital of Sun Yat-Sen University, Guangzhou, 510630 China; 4 The First Affiliated Hospital of Guangdong Pharmaceutical University, School of Clinical Medicine of Guangdong Pharmaceutical University, Guangzhou, 510008 Guangdong China; 5 Department of Cardiovascular Surgery, Guangdong Cardiovascular Institute, Guangdong Provincial Key Laboratory of South China Structural Heart Disease, Guangdong General Hospital, Guangdong Academy of Medical Sciences, Guangzhou, 510080 China

**Keywords:** Dilated cardiomyopathy, Metabonomic analysis, Mass spectrometry analysis, Purine metabolism reprogramming

## Abstract

**Background:**

The crosstalk of purine biosynthesis and metabolism exists to balance the cell energy production, proliferation, survival and cytoplasmic environment stability, but disorganized mechanics of with respect to developing heart failure (HF) is currently unknown.

**Methods:**

We conducted a multi-omics wide analysis, including microarray-based transcriptomes, and full spectrum metabolomics with respect to chronic HF. Based on expression profiling by array, we applied a bioinformatics platform of quantifiable metabolic pathway changes based on gene set enrichment analysis (GSEA), gene set variation analysis (GSVA), Shapley Additive Explanations (SHAP), and Xtreme Gradient Boosting (XGBoost) algorithms to comprehensively analyze the dynamic changes of metabolic pathways and circular network in the HF development. Additionally, left ventricular tissue from patients undergoing myocardial biopsy and transplantation were collected to perform the protein and full spectrum metabolic mass spectrometry.

**Results:**

Systematic bioinformatics analysis showed the purine metabolism reprogramming was significantly detected in dilated cardiomyopathy. In addition, this result was also demonstrated in metabolomic mass spectrometry. And the differentially expressed metabolites analysis showing the guanine, urea, and xanthine were significantly detected. Hub markers, includes *IMPDH1*, *ENTPD2*, *AK7*, *AK2*, and *CANT1*, also significantly identified based on XGBoost, SHAP model and PPI network.

**Conclusion:**

The crosstalk in the reactions involved in purine metabolism may involving in DCM metabolism reprogramming, and as coregulators of development of HF, which may identify as potential therapeutic targets. And the markers of *IMPDH1*, *ENTPD2*, *AK7*, *AK2*, and *CANT1*, and metabolites involved in purine metabolism shown an important role.

**Supplementary Information:**

The online version contains supplementary material available at 10.1186/s12967-022-03739-3.

## Introduction

The heart is a blood-pumping organ with the highest energy demand in the body. In a normal heart, 60%–80% of energy-producing substances are free fatty acids (FFA), while 10%–20% are derived from glucose, acetone, lactic acid, and ketone bodies [1]. Heart failure, the end stage of multiple cardiovascular diseases, is a development process with cardiac remodeling as the core, in which multiple factors such as hemodynamics, neurohormones, genetic factors, and energy metabolism participate jointly [2]. Among these, abnormal energy metabolism is not only the direct manifestation of heart failure symptoms, but also one of their pathological bases. Normal myocardial energy metabolism comprises the following three steps [3–5]: (1) the utilization of substrates, (2) the oxidative phosphorylation of mitochondrial respiratory chain, and (3) the transport and utilization of ATP. Problems arising in any of the three steps would cause disorders of myocardial energy metabolism.

Heart failure often manifests as energy deficiency and mitochondrial oxidative damage due to changes in energy substrates. Metabolites associated with glucose, lipid, and amino acid metabolism processes are abnormal in heart failure. In an early stage of the disease, fatty acid (FA) oxidation (FAO) appears to be normal or slightly higher. With progression of the disease, FAO is impaired, and glucose is then preferentially used as a substrate for energy metabolism, known as the “cardiac metabolic reprogramming” [6]. In 2004, van Bilsen et al. put forward the concept of metabolic remodeling of the failing myocardium, which argued that when heart failure occurs, myocardial structure and cell metabolism are both disordered, causing cardiac dysfunction, and changes in cardiac energy metabolism give rise to severe heart failure [7]. In addition, Guo et al. investigated the mechanisms of heart failure using a metabolomics technique based on ultra-performance liquid chromatograph quadrupole time-of-flight mass spectrometry (UPLC/TOF–MS). They identified 13 metabolites in the serum as potential biomarkers of heart failure, and these compounds were mainly associated with inflammation, energy metabolism disorders, and amino acid disorders [8]. Li et al. established a rat model of chronic heart failure (CHF) and identified 23 metabolites related to CHF with non-targeted metabonomics. Their results showed that the metabolism of branched chain amino acids (BCAA) in the heart of rats with heart failure was significantly inhibited [9]. Li et al. selected 27 healthy, 22 stage B1, 18 stage B2 pre clinical MMVD dogs with mucinous mitral valve disease and 17 MMVD dogs with congestive heart failure (CHF) history for metabonomic analysis. They found that there were 173 known metabolites of different concentrations among the four groups, of which 40% were amino acids and 30% were lipids, revealing changes in energy metabolism and amino acid metabolism during the occurrence and development of MMVD and CHF [10]. Li et al. used the method of metabonomics and 16S rRNA sequencing to analyze the fecal metabolism profile and intestinal microbial composition of H-HF rats, and found that the intestinal microbial composition of H-HF rats had changed significantly, the mycelium/Bacteroid (F/B) ratio increased, and the number of bacteria in rhamnoideae, lactobacilliaceae, and lactobacilliaceae decreased. The levels of 17 genera and 35 metabolites changed significantly and were identified as potential biomarkers of H-HF. Correlation analysis showed that there was a strong correlation between specific altered genera and altered fecal metabolites. The reduction of short chain fatty acid (SCFA) producing bacteria and trimethylamine N-oxide (TMAO) may be a significant feature of H-HF [11]. Furthermore, Juho Heliste et al. included Finnish patients with heart failure to screen out a new genetic variation related to heart failure, and identified a new variation for function through in vitro and in vivo studies. This study suggests the role of TRIM55 gene polymorphism in heart failure susceptibility [12]. Vilela et al. [13] found that uncoupling protein 2 (UCP2), a proton transporter located in the inner mitochondrial membrane, can transport H^+^ from the outer side back to the inner side of the membrane. This reduces the electrochemical gradient of H^+^ across the membrane formed during substrate oxidation, and decouples oxidative phosphorylation of the respiratory chain from ATP synthesis. As a result, the energy released from H^+^ oxidation is converted into heat, and ATP production is diminished. If UCP2 expression is upregulated in smooth muscle tissue, it can further mediate a reduction in ATP production, increasing myocardial energy metabolism disorders and thereby aggravating heart failure. Therefore, UCP2 is considered to be a “ruler” of myocardial cell metabolism, which can sense changes in the metabolism–energy state. UCP2 influences multiple steps of substrate metabolism to modulate the process of glycolysis, glucose uptake, and energy production, regulate the efficiency of oxidative phosphorylation, and maintain the balance of energy supply and demand [13]. Kim et al. and Fry et al. have all demonstrated that beta3 adrenoceptor (beta3-AR) agonists inhibit adipocyte differentiation by downregulating gene expression levels of peroxisome proliferator-activated receptor (PPAR) and adipocyte FA-binding protein (aP2), thereby causing the reversion of myocardial energy metabolism back towards fetal energy metabolism [14, 15]. Other researchers have proposed that cardiac beta3-AR activates the extracellular signal-regulated kinase/mitogen-activated protein kinase (Erk-MAPK) pathway through phosphorylation, which downregulates PPAR-alpha expression or activity; subsequently, FAO is impaired and metabolic remodeling is induced. This may be another mechanism by which beta3-AR mediates negative inotropic effects through influencing myocardial cell metabolism, but it still needs to be verified. Perilipin 5 (Plin5) plays a role in bidirectional regulation of lipid metabolism balance during energy metabolism in myocardial cells. Plin5 binds to adipose triglyceride lipase (ATGL), thereby inhibiting lipid dissolution and facilitates FA storage. When stress or heart failure occurs, protein kinase A (PKA) phosphorylates and activates Plin5, promotes the release of ATGL from the complex, and triggers ATGL activity, thus accelerating FA degradation and participating in metabolic reprogramming of myocardial cells [16].

Metabolomics is a scientific method emerging after genomics and proteomics in recent years, and it constitutes an essential part of systems biology. Metabonomic analysis can detect small-molecule metabolites (including lipids, carbohydrates, and amino acids), and these indicators are further analyzed using multivariate statistical methods, such as principal component analysis (PCA), partial least squares discriminant analysis (PLS-DA) [17], and orthogonal partial least squares discriminant analysis (OPLS-DA) in order to determine the corresponding biomarkers and elaborate the mechanisms of disease pathogenesis and associated molecular pathways. Identification of novel biomarkers can predict disease progression and guide individualized treatment [18]. Various physiological reactions catalyzed by gene encoding enzymes and their interaction systems can be reflected through metabolic networks [19]. In the present study, an integrated analysis strategy based on non-targeted metabolomics and transcriptomics was used to analyze the metabolic profile of patients with heart failure, and patients without heart failure were included as a control group. The aim of the study was to explore systemic metabolic changes in heart failure and the involved pathways of metabolic disorders, and to search for novel markers and therapeutic targets for heart failure. The findings could provide new thoughts for clinical diagnosis and treatment of heart failure.

## Methods

### Transcriptional expression profiling analysis

The CEL raw microarray data of GSE57338 [20], GSE19303 [21], and GSE120895 [22] were downloaded from NCBI GEO database (https://www.ncbi.nlm.nih.gov/geo/). In order to remove the interference of pathological phenotypes of other heart diseases, only the DCM and control samples before any treatment were selected. In GSE57338, annotated by platforms of GPL11532 (Affymetrix Human Gene 1.1 ST Array), the left ventricle (LV) myocardium of DCM and non-failing controls were included, and ischemic myocardium excluded. In GSE19303, annotated by platforms of GPL570 (Affymetrix Human Genome U133 Plus 2.0 Array), the LV endomyocardial biopsies of DCM patients before the treatment of immunoglobulin substitution (IA/IgG) and controls were selected. Additionally, through literature reading and data set unit filtering, we find that the datasets of GSE19303 and GSE120895 actually come from the same research team (Sabine Ameling’s group; University Medicine Greifswald, Greifswald, Germany). In order to avoid repeated application of data, we select the GSE19303 dataset for the subsequent analysis.

**Fig. 1:**
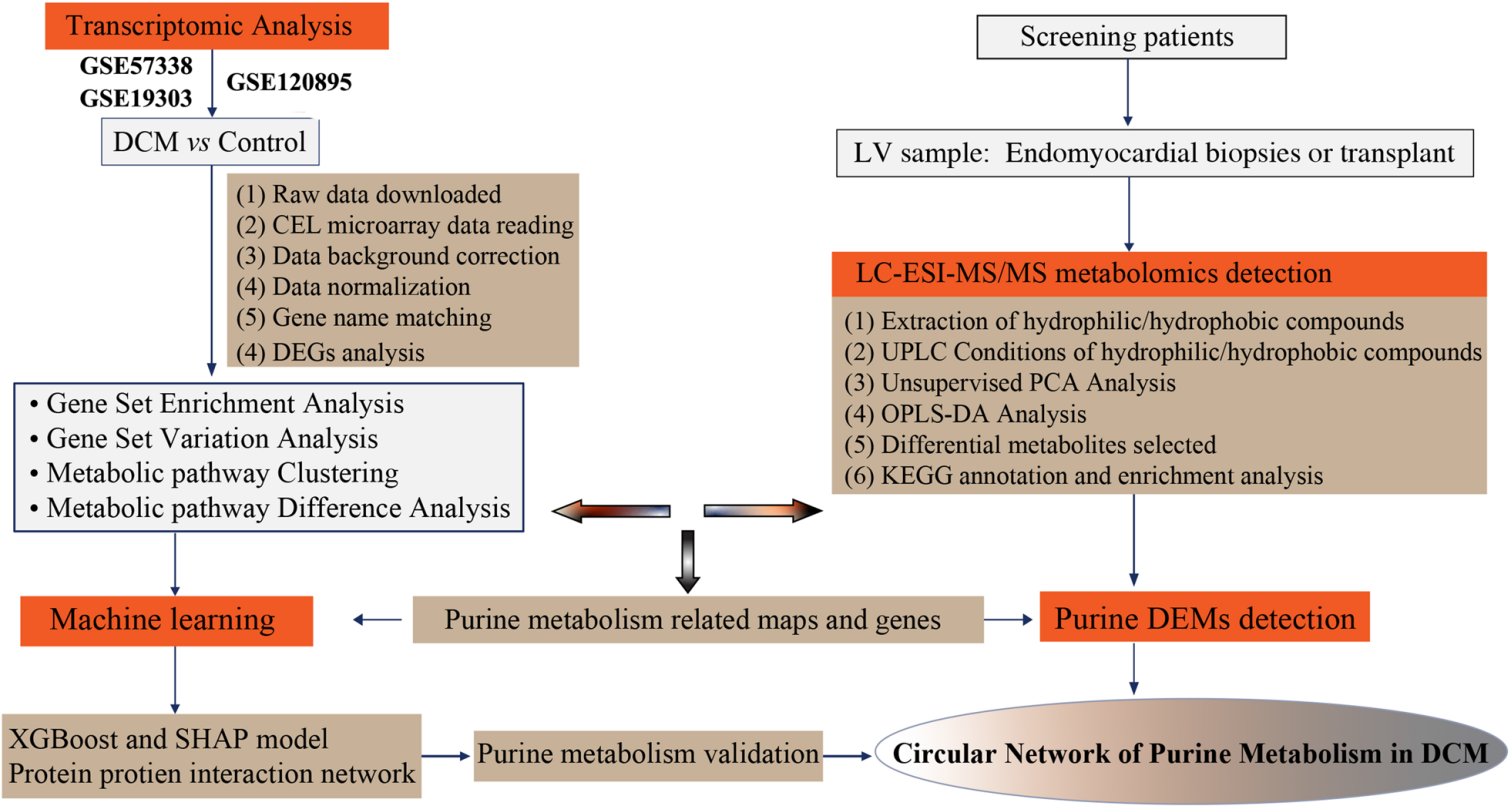
Flowchart overview of this study

Here, microarray data processing as following [23]: (1) raw data (CEL files) were downloaded; (2) “Affy” or “Oligo” algorithm were used for raw CEL fluorescence microarray data reading; (3) “RMA” algorithm was applied for data background correction, normalization, gene name matching, and missing value handling; (4) “LIMMA” method was used for differential gene expression analysis. The criteria for selecting differentially expressed genes: log2 fold change ≥ 1.5; Benjamini-Hochberg (B-H) adjusted *P*-value < 0.05. The analysis process is shown in Fig. 1.

### Gene set enrichment analysis and gene set variation analysis

We conducted a gene-set enrichment analysis (GSEA; http://software.broadinstitute.org/gsea/index.jsp) of based on “clusterProfiler” and “AnnotationHub” algorithms [24, 25]. Here, the algorithmic parameters of the number of genes in the minimum gene set is 10, as well as 500 in the largest gene set. The terms with B-H corrected *P*-value < 0.05 with the permutation test number is 1000 were considered as significantly enriched pathway.

Additionally, gene set variation analysis (GSVA) is a non-parametric unsupervised analysis method that is used to evaluate the results of gene set enrichment of microarray or RNA-seq. By integrating the Molecular Signatures Database (v7.5.1; http://www.gsea-msigdb.org/gsea/msigdb/index.jsp), the gene list of pathways was collected [26, 27]. The GSVA and the GSEABase packages were used for standardized scoring of the gene sets for each cell.

### Participant inclusion

The study recruited consecutive patients who presented to the Department of Cardiovascular Surgery at the Guangdong General Hospital and Guangdong Academy of Medical Sciences (Approved No. of Ethics Committee: No. GDREC2020162H(R1)) in June 2019–July 2021. According to the 2016 ESC Heart Failure Guidelines, the clinical diagnostic criteria for DCM are objective evidence of decreased ventricular dilatation and myocardial contraction functions [28]: ① left ventricular end-diastolic diameter > 5.0 cm (female) or > 5.5 cm (male) (or > 117%, i.e., 2SD + 5% of the predicted value of age and body surface area); ② left ventricular ejection fraction < 45% (Simpsons method) and left ventricular short-axis shortening rate (LVFS) < 25%; and ③ excluding hypertension, valvular heart disease, congenital heart disease, or ischemic heart disease. Samples of the control group were obtained from myocardial biopsies of three relatively healthy participants. The patient exclusion criteria were as follows: (1) the patient was aged less than 18 years old; (2) the patient had systemic disease that affected the metabolism, such as malignant tumor, autoimmune disease, endocrine disease, or end-stage renal insufficiency; (3) the patient had blood-borne infectious disease, such as acquired immunodeficiency syndrome, hepatitis B, or hepatitis C; and (4) the patient lacked tissue samples. All participants signed an informed consent form. The study was approved by the hospital ethics committee and complied with the 1975 Declaration of Helsinki (2000 revision) [29].

### Mass spectrometry-based metabolomic profiling

The LC–ESI–MS/MS system (UPLC, ExionLC AD, https://sciex.com.cn/; MS, QTRAP® System, https://sciex.com/)30 were measured in our mass spectrometry study. A triple quadrupole-linear ion trap mass spectrometer (QTRAP; QTRAP® LC–MS/MS System), equipped with an ESI Turbo Ion-Spray interface, were acquired via the LIT and triple quadrupole (QQQ) scans. Additionally, the positive and negative ion mode analyzed via Analyst 1.6.3 software (Sciex) [31]. Quality control was performed with a mixture of the sample extracts. Quality control samples were inserted into analytical samples, once every ten analytical samples, to monitor the repeatability of measurement under the same operating conditions. The total ion current (TIC) chromatogram and multiple reaction monitoring (MRM) multi-peak chromatogram were obtained. A triple quadrupole mass spectrometer was used to select the characteristic ion of each substance. The signal intensity (counts per second) of the characteristic ion was acquired in the detector. With the mass spectrometer output files of samples, MultiQuant software was used for chromatographic peak integration and correction. Chromatographic peak area represents the relative content of the corresponding substance [32]. We exported and saved all chromatographic peak area integration data. To compare the content of each detected metabolite in different samples, we corrected the chromatographic peak of each metabolite in different samples based on the retention time and peak type of the metabolite, to ensure the accuracy of qualification and quantification.

### Orthogonal partial least squares-discriminant analysis

With the metabolomic data collected above, we could carry out metabolite identification and sample data quality control analysis, select differential metabolites, and perform function prediction and analysis of metabolites in samples. Orthogonal partial least squares-discriminant analysis (OPLS-DA), which combines orthogonal signal correction and PLS-DA, decomposes the matrix information of independent variables into dependent variables-related and -unrelated information [32]. This method enables maximization of intergroup differences, facilitating the identification of differential metabolites, and filters out unrelated differences to further identify differential variables, enriching differential analysis results. Based on “MetaboAnalystR” R package, variable importance in projection (VIP) from the OPLS-DA model was used to preliminarily select differential metabolites between groups [33].

The data was log transform (log2) and mean centering for further differential metabolite analysis. And a permutation test with 200 permutations was applied to eliminate the data overfitting. We defined metabolites with VIP ≥ 1.0 and absolute Log2FC (fold change) >  = 1 as the significantly differential metabolites (i.e., if the ratio of a metabolite in the tumor group to that in the control group was ≥ 2 or ≤ 0.5, the difference was considered as statistically significant).

### Functional annotation and enrichment analysis of differential metabolites

Based on the Kyoto Encyclopedia of Genes and Genomes (KEGG) Compound database (http://www.kegg.jp/kegg/compound/) and KEGG Pathway database [34], we’re subquentlly annotated metabolites, analyzed the interactions and mapped to the pathways of potential metabolic pathways, including the carbohydrate, nucleotide, and amino acid metabolism and organic substance biodegradation, and carried out comprehensive annotation of enzymes for a series of reactions. For pathway enrichment, the hypergeometric test’s *P* value < 0.05 was considered as statistically significant.

Traditional enrichment analysis based on hypergeometric distribution is mainly fit for differential metabolites with marked upregulation or downregulation, tending to miss some metabolites without significant differential expression but with important biological significance. Metabolic set enrichment analysis (MSEA) enables enriching metabolomic data into a series of preset metabolic sets, without pre-specifying the threshold of differential metabolites [35]. We used MSEA to identify metabolic sets with significant differences. *P* value < 0.05 for pathway enrichment was considered as statistically significant.

### Hub markers detection based on machine learning and PPI network

The expression level of purine metabolism gene list was extracted from GSE120895 dataset. The gene expression value prediction algorithm based on XGBoost is superior to the traditional machine learning algorithm and D-GEX algorithm [36]. And with this method, the hub genes were identified. A total of 221 samples were selected, which included 137 control and 84 DCM samples. And then we’re split the dataset: 75% for training, 25% for validation. Bayesian optimization algorithm and learning framework based on Boost tree model have optimized the important super parameters and parameters of XGBoost, greatly improving the stability, prediction accuracy and calculation efficiency of the model. The prediction results of the model are explained based on Shapley Additive ExPlans (Sharp) method [37].

$$ {\$}\left(\mathrm{\varphi }\right)={\sum }_{i=1}^{n}loss\left(Xixi\right)+{\sum }_{m=1}^{m}\Omega \left(fk\right)$$

The training loss is loss, the complexity of the tree is Ω(f), and the number of trees is k in this formula [38]. The XGBoost fine-tuned parameters is: learning rate = 0.05, gamma = 0.009, subsample = 0.85, colsample bytree = 0.8, max depth = 8, and num boost round (boosting iterations) = 500.

SHAP is the only consistent and locally accurate feature attribute method based on expectations. This technology can explain the feature importance score in the complex training model, and propose an interpretable prediction for the test sample. SHAP values are proposed to uniformly measure feature importance because importance values (fi) were assigned to each feature which exemplifies the effect of including features in the model predictions. The SHAP value was calculated as follows in cooperative game theory [39]:where F denotes the set of all features and S denotes the subset of all features obtained from F after the removal of the ith feature. Then, the two models fsU{i} and fs are retrained and the predictions of these two models are compared with the current input fsU{i}(xsU{i})-fs(xs), where xs denotes the value of the input features in the set S. To estimate  from 2^|F|^ differences, the SHAP method approximates the Shapley values by performing Shapley sampling or Shapley quantitative influence.

Similarly, as a conventional calculation method, the calibration curve of AUC, accuracy, sensitivity, and specificity of the model for the test set were drawn. The top 10 with higher SHAP value presented as the hub gene explained by black-box ML models (MLGs).

After extract the top 15 important genes from the SHAP model, STRING online database (version 11.0; https://string-db.org/) [40], a protein–protein interaction (PPI) network was conducted with the threshold of 0.4. The PPI network was constructed utilizing Cytoscape software (version 3.8.2; https://cytoscape.org/). And the degree of each node in each cluster based on Maximal Clique Centrality (MCC) method were calculated via the CytoHubba software [40]. The advantages of MCC is can capture the dynamic characters following the time series, and then clustered these nodes based on iterative computation. For the network, top 10 gene with the highest degree in the PPI network was screened out as the hub gene with important biological function (BFGs). In this plot, we overlap the top 10 BFGs and MLGs to detect the important features, which has the characteristics of both biological function and classification and prediction ability of ML model. Finally, the GSVA- normalized map score of purine pathways and RMA-normalized hub gene’s expression level of GSE120895were calculate dataset for the external validation.

## Results

### Transcriptional expression profiling analysis

**Fig. 2:**
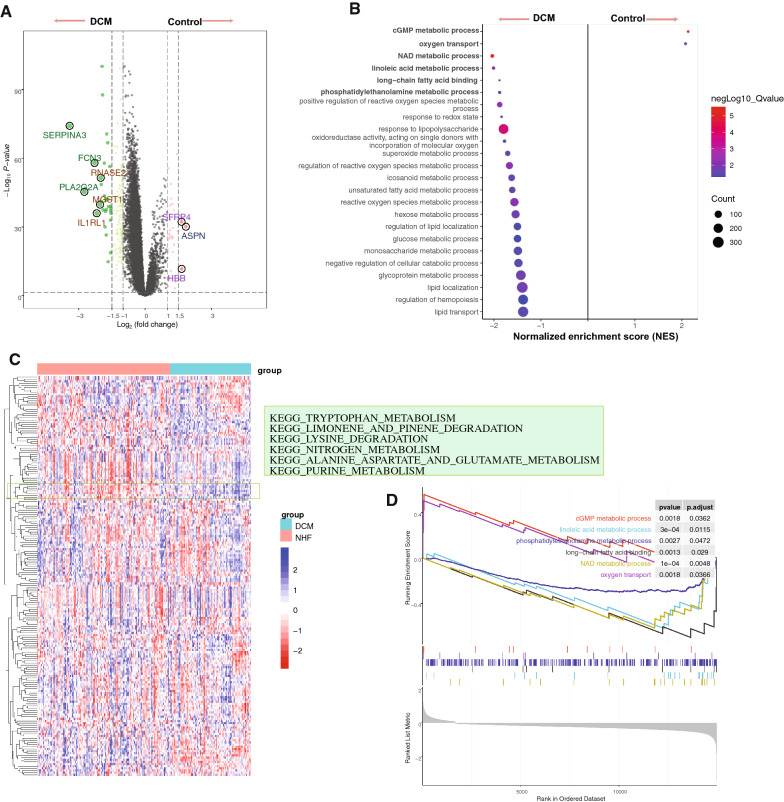
The differential expression and functional pathway analysis in GSE57338 dataset. **A** The volcano map showing the differential expressed genes between the control and DCM samples. Green dots are down-regulated DEGs and red dots are up-regulated DEGs. **B** The GSEA analysis shown the maps of cGMP metabolic process, oxygen transport, NAD metabolic process, linoleic acid metabolic process, long-chain fatty acid binding, and phosphatidylethanolamine metabolic process were significantly enriched. The color bar indicates the corrected p value of the enrichment pathway. The size of the dot indicates the number of enriched genes. **C** The heatmap showing the patterns of the GSVA score of the KEGG pathway, and the metabolism- related maps were significantly identified. **D** The purine metabolism- related maps were detected based on GSEA analysis

Based on DE analysis, a total of 177 DEGs were detected (included 143-downregulated and 34-upregulated). Figure 2A shown the top DEGs in GSE57338 dataset. And the list of DEGs shown in Additional file 1: Table S1.

### Gene set enrichment analysis and gene set variation analysis

The GSEA analysis showed that the metabolic related pathways were identified. And the results shown that the cGMP metabolic process (Normalized enrichment score (NES) = 2.11,adjusted p-value = 0.0362, enriched gene = 3), linoleic acid metabolic process (NES =  − 1.97, adjusted p-value = 0.0115, enriched gene = 13), phosphatidylethanolamine metabolic process (NES =  − 1.87, adjusted p-value = 0.0472, enriched gene = 4), long-chain fatty acid binding (NES =  − 1.89, adjusted p-value = 0.029, enriched gene = 5), NAD metabolic process (NES =  − 2.00, adjusted p-value = 0.0048, enriched gene = 14), and oxygen transport (NES = 2.09, adjusted p-value = 0.0366, enriched gene = 3) were significantly identified (Fig. 2B, D).

Of the GSVA-normalized pathway score detection, the maps, includes purine metabolism, alanine aspartate and glutamate metabolism, cysteine and methionine metabolism, sphingolipid metabolism, O-glycan biosynthesis, and tryptophan metabolism, where significant difference were detected between the control and DCM samples based on “LIMMA” powerful different analysis (Fig. 2C and Table 1). Here, integrating the above differential pathway analysis results, we found that the purine metabolic pathway, an interesting metabolic change, may play an important regulatory role in the occurrence and process of DCM heart failure. The list of pathway analysis results following the GSEA and GSVA algorithms were presented in Additional file 2: Table S2 and Additional file 3: Table S3, respectively.

**Table 1:** The differential analysis of metabolic pathways based on gene set variation analysis

Terms	t	Adj.P.Val	B
KEGG_PURINE_METABOLISM	9.02	6.11E−16	29.98
KEGG_ALANINE_ASPARTATE_AND_GLUTAMATE_METABOLISM	8.65	3.65E−14	25.15
KEGG_CYSTEINE_AND_METHIONINE_METABOLISM	− 9.29	6.14E−16	29.38
KEGG_SPHINGOLIPID_METABOLISM	− 8.41	1.39E−13	23.67
KEGG_O_GLYCAN_BIOSYNTHESIS	− 8.03	1.41E−12	21.25
KEGG_TRYPTOPHAN_METABOLISM	7.58	1.78E−11	18.52

### Mass spectrometry and orthogonal partial least squares-discriminant analysis

**Fig. 3:**
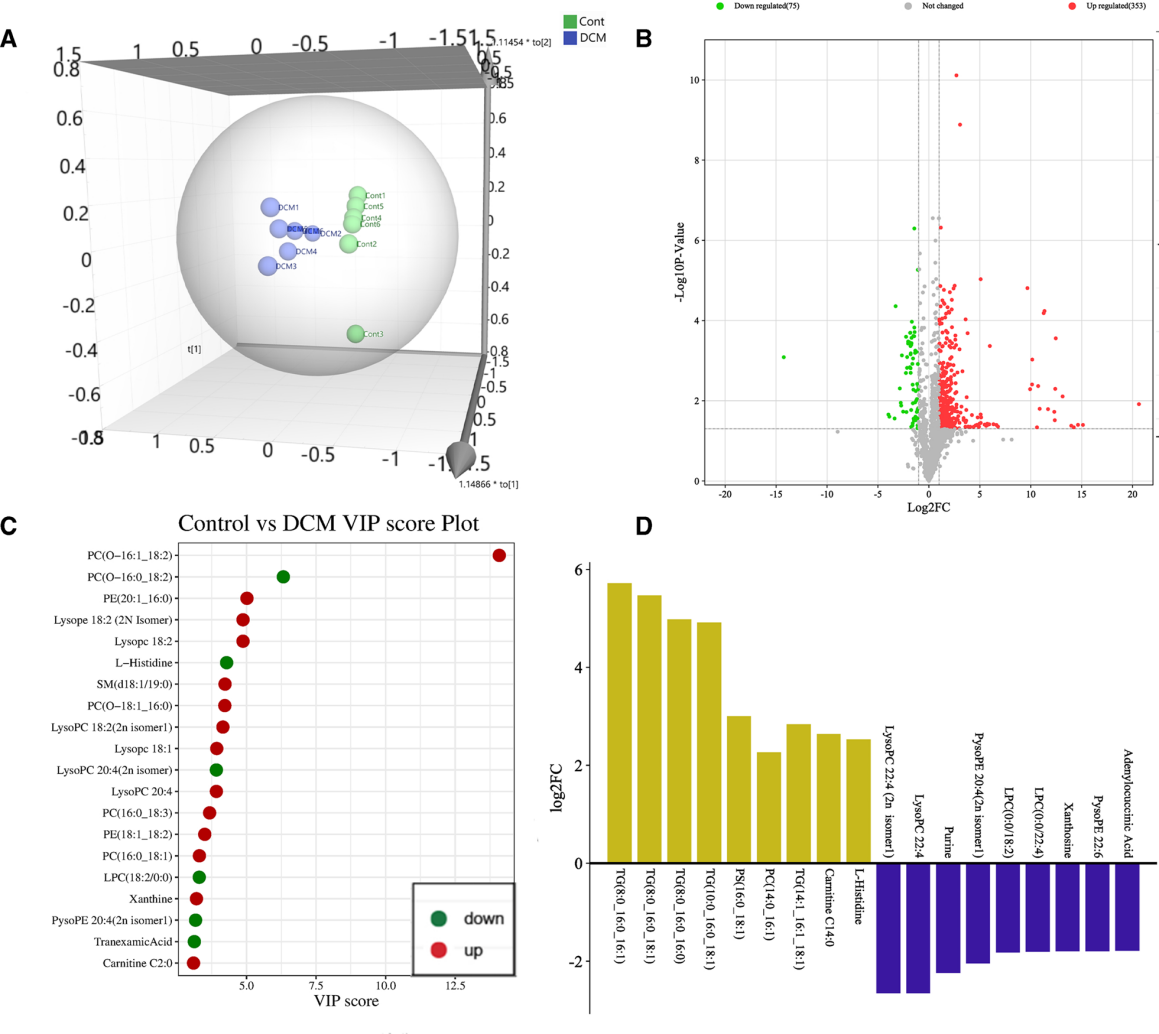
LC–ESI–MS/MS differentially expressed metabolites (DEMs) detection. **A** The clustering plot showing a significant difference among the DCM and control group basing on the OPLS-DA model. **B** The volcano plot shows the difference in the expression level of metabolites between the DCM and control groups, and the statistical significance of the DEMs. **C** The VIP values of different metabolites. The red dots representing the up-regulated DEMs, and the green points representing the down-regulated DEMs. **D** After the qualitative and quantitative analysis of metabolites, log2 transformed, and the change of difference of DEMs is presented. Red is the up-regulated DEMs, and green is the down-regulated DEMs

Here, 12 patients were included in our study (6 control samples from myocardial biopsy in patients with suspected myocarditis, 6 DCM samples from heart transplantation). Based on extensive targeting technology, 1474 metabolites were detected. OPLS-DA combining the orthogonal signal correction (OSC) and partial least squares discriminant analysis (PLS-DA) methods, which can effectively extract the main information of variables with less correlation, and filter the difference variables by removing irrelevant differences. According to the analysis of OPLS-DA model, Fig. 3A shows the scores of control and DCM groups, suggesting that there is a significant difference between the two groups. Based on OPLS-DA model, the VIP score preliminarily screen the metabolites with different among the control and DCM samples. In results, according to the criterion of differentially expressed metabolites (DEMs), the 75 metabolites defined as significantly down-regulated, and 353 metabolites were up-regulated (Fig. 3B). And Fig. 3C shown the significant DEMs with the higher VIP score, which includes the PC(O-16:0_18:2), L-Histidine, LysoPC 20:4(2n isomer), LPC(18:2/0:0), PysoPE 20:4(2n isomer1), Tranexamic Acid, PC(O-16:1_18:2), PE(20:1_16:0), Lysopc 18:2, Lysope 18:2 (2 N Isomer), SM(d18:1/19:0), PC(O-18:1_16:0), LysoPC 18:2(2n isomer1), Lysopc 18:1, LysoPC 20:4, PC(16:0_18:3), PE(18:1_18:2), PC(16:0_18:1), Xanthine and Carnitine C2:0. Additionally, the top up or down-regulated DEMs also presented in Fig. 3D (Up-regulated: TG(8:0_16:0_16:1), TG(8:0_16:0_18:1), TG(8:0_16:0_16:0), TG(10:0_16:0_18:1), PS(16:0_18:1), PC(14:0_16:1), TG(14:1_16:1_18:1), Carnitine C14:0 and L-Histidine); Down-regulated: LysoPC 22:4 (2n isomer1), LysoPC 22:4, Purine, PysoPE 20:4 (2n isomer1), LPC(0:0/18:2), LPC(0:0/22:4), Xanthosine, PysoPE 22:6 and Adenylocuccinic Acid). And the metabolite expression profiles and DEMs results shown in Additional file 4: Table S4.

### Functional annotation and enrichment analysis of differential metabolites

**Fig. 4:**
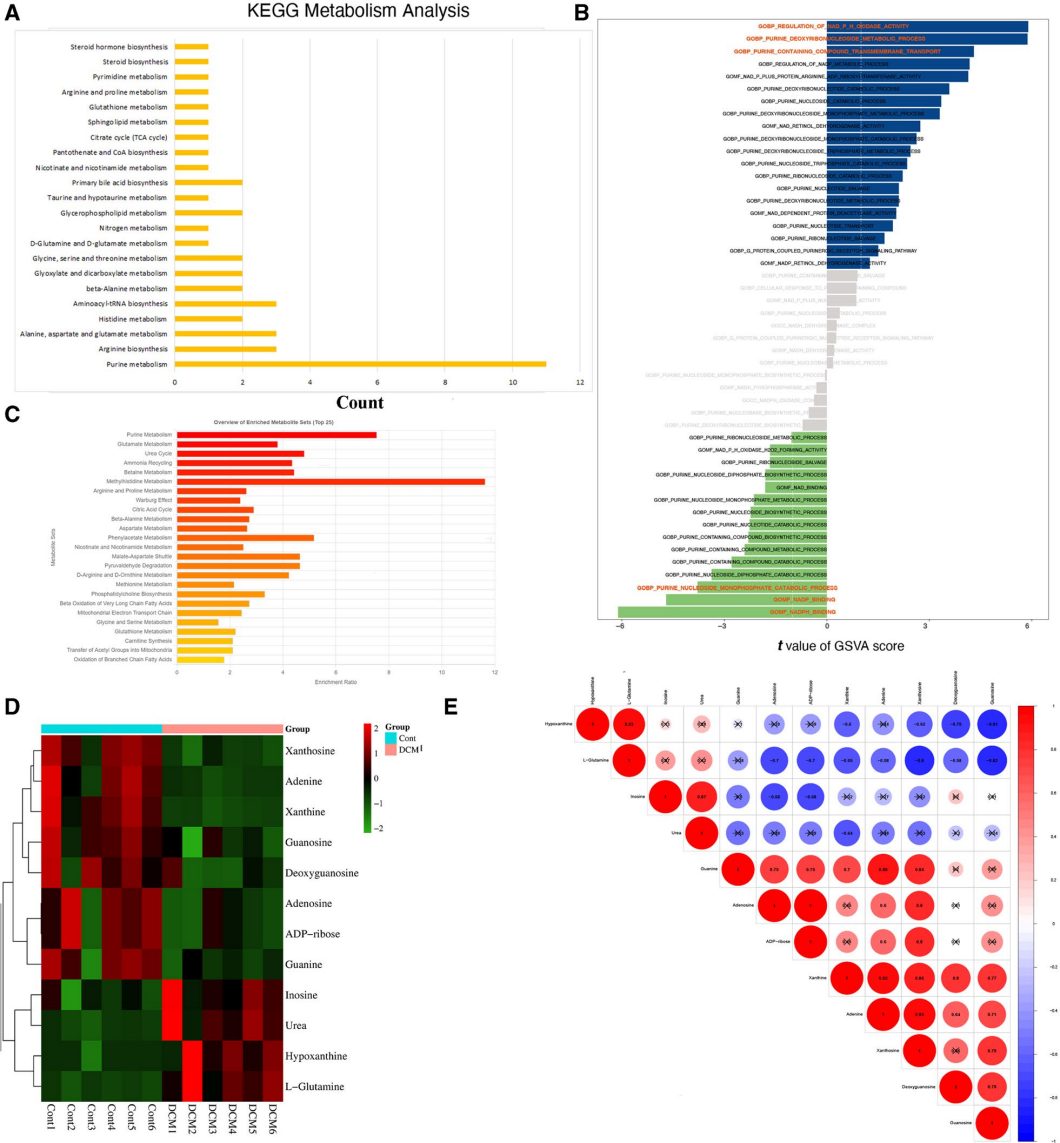
The functional enrichment and correlation analysis among the DEMs. **A** The KEGG metabolism analysis results were detected, and the abscissa is the number of DEMs enriched in the corresponding maps, and the proportion of DEMs also calculated. **B** The bar plot showing the patterns of the GSVA score of the purine metabolism, and the difference detected by LIMMA algorithm. **C** Maps of purine metabolism, histidine metabolism, and beta-Alanine metabolism were significantly identified based on MSEA analysis. **D** The heatmap shown the purine- related metabolites relative expression levels. **E** The correlation heatmap presenting the interaction relationship basing on partial correlation network analysis

The biological DEMs interacts in organisms to form different pathways. Figure 4A shown the annotation results of the DEMs are classified according to the metabolic pathway in KEGG. Here, the Purine metabolism (DEMs = 11, rate = 16.92%), Arginine biosynthesis (DEMs = 3, rate = 21.43%),

Alanine, aspartate and glutamate metabolism (DEMs = 3, rate = 10.71%), Histidine metabolism (DEMs = 2, rate = 12.5%), Aminoacyl-tRNA biosynthesis (DEMs = 3, rate = 6.25%), beta-Alanine metabolism (DEMs = 2, rate = 9.52%), et al. And the maps including purine metabolism, glutamate metabolism, et al. were primarily enriched (Fig. 4C).

Of the purine pathway, the different analysis of GSVA score shown the maps of GOBP: regulation of NAD-P–H oxidase activity (t = 5.90, adjusted P-Value = 2.41E-07, B = 9.22), GOBP: purine deoxyribonucleoside metabolic process(t = 5.88, adjusted P-Value = 2.41E-07, B = 9.12), and GOBP: purine containing compound transmembrane transport (t = 5.90, adjusted P-Value = 2.41E-07, B = 9.22)were up-regulated; while the pathway of GOBP: purine nucleoside monophosphate catabolic process(t = -3.79, adjusted P-Value = 0.0012, B = 0.092), GOMF:NADP binding(t =  − 4.70, adjusted P-Value = 5.33E-05, B = 3.67), and GOMF:NADPH binding (t =  − 6.11, adjusted P-Value = 2.04E-07, B = 10.34) were down-regulated. The GSVA score of purine metabolism related pathway were presented in Fig. 4B, Additional file 5: Table S5.

Finally, the expression level of 12 purine DEMs, includes hypoxanthine, guanine, inosine, urea, L-glutamine, adenosine, adenine, deoxyguanosine, ADP-ribose, xanthine, xanthosine, and guanosine, shown in Fig. 4D. In addition, Fig. 4E inspecting the correlation of 12 purine DEMs based on metabolites expression level. And Table 2 presenting the differential results of metabolites.

**Table 2:** Results of differential analysis of purine metabolites

Compounds	Formula	Class.II	CAS	VIP	Log2FC	Type
Guanine	C5H5N5O	Nucleotide and Its metabolomics	73–40–5	1.98	1.78	Up
Urea	CH4N2O	Amines	57–13–6	1.85	1.95	Up
Xanthine	C5H4N4O2	Nucleotide and Its metabolomics	69–89–6	0.37	− 1.45	Down
L-Glutamine	C5H10N2O3	Amino acids	56–85–9	1.26	0.34	Insig
Adenosine	C10H13N5O4	Nucleotide and Its metabolomics	58–61–7	1.67	0.72	Insig
Xanthosine	C10H12N4O6	Nucleotide and Its metabolomics	146–80–5	0.38	− 0.07	Insig
Hypoxanthine	C5H4N4O	Nucleotide and Its metabolomics	68–94–0	1.30	0.34	Insig
Inosine	C10H12N4O5	Nucleotide and Its metabolomics	58–63–9	0.47	0.09	Insig
Deoxyguanosine	C10H13N5O4	Nucleotide and Its metabolomics	961–07–9	0.85	0.35	Insig
Guanosine	C10H13N5O5	Nucleotide and Its metabolomics	118–00–3	0.75	0.25	Insig
ADP-ribose	C15H23N5O14P2	Nucleotide and Its metabolomics	20762–30–5	1.05	0.41	Insig
Adenine	C5H5N5	Nucleotide and Its metabolomics	73–24–5	0.51	0.14	Insig

### Hub markers detection based on machine learning and PPI network

**Fig. 5:**
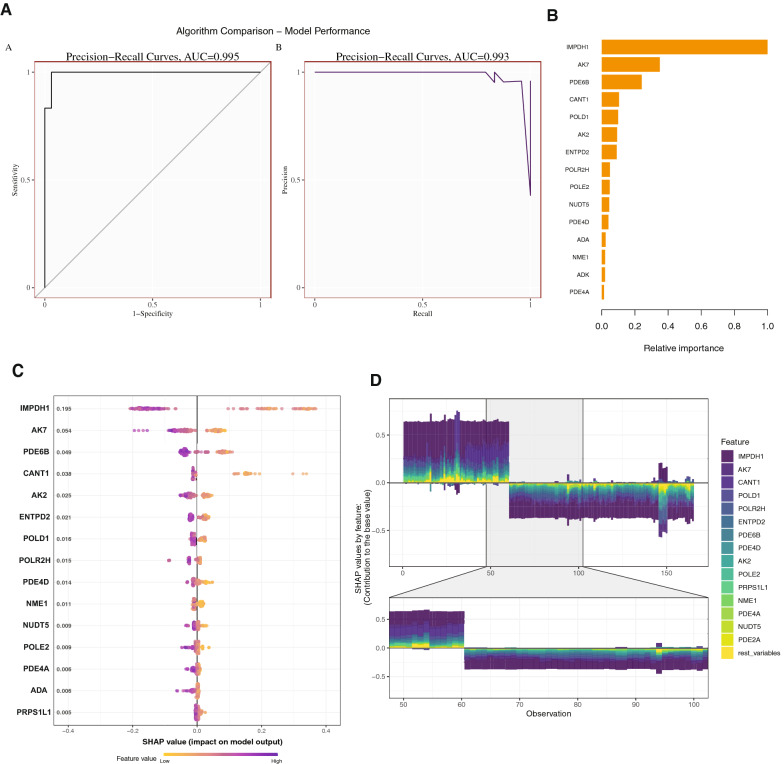
The hub markers detection based on XGBoost and SHAP model. **A** The AUC curves showing the prediction performance of sensitivity, specificity, precision, and recall based on the gene’s expression of purine metabolism pathway. **B** The bar plot showing the top 15 relative importance features in our ML model. **C** The SHAP summary plot presenting the SHAP value (relative impact on ML model) of top 15 relative importance features. **D** The SHAP force plot showing the hub gene contribution to the base value in SHAP model

The receiver operating characteristic (ROC) curves shown the outstanding efficacy of the XGboost model for outcome prediction (AUC = 0.995, 95% confidence interval [CI] 0.993–0.997), and the recall results (AUC = 0.993, 95% CI 0.992–0.995) (Fig. 5A). To identify the important features, the SHAP summary plot the top 15 features of the of XGboost model (Fig. 5B), and SHAP value of the features impact on XGboost model also depicted (Fig. 5C). The Fig. 5D presented the distribution of SHAP values for the top 15 observation in the form of force plot, and thus reflecting the sum of each predictor’s attributions.

**Fig. 6:**
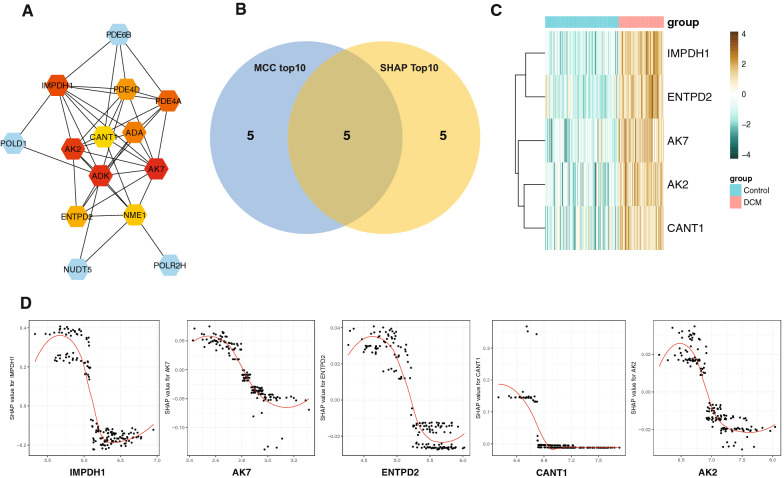
The hub marker detection based on protein–protein network analysis. **A** The hub genes with the highest MCC value were detected in the PPI network based on the CytoHubba algorithm. **B** The venn plot presenting the co-hubs among the top markers in PPI network and SHAP model. **C** The heatmap showing the hub marker expression level in GSE120895 dataset. **D** This plot depicts presenting the relationship among the hub gene’s expression levels and SHAP values

In addition, the hubs includes inosine-5′-monophosphate dehydrogenase 1 (*IMPDH1*; MCC = 260, Degree = 9, Clustering Coefficient = 0.56, Radiality = 3.93), cAMP-specific 3′,5′-cyclic phosphodiesterase 4D (*PDE4D*;MCC = 138,Degree = 7, Clustering Coefficient = 0.71, Radiality = 3.71), cAMP-specific 3′,5′-cyclic phosphodiesterase 4A (*PDE4A*; MCC = 246, Degree = 7,ClusteringCoefficient = 0.76, Radiality = 3.71), soluble calcium-activated nucleotidase 1 (*CANT1*;MCC = 14, Degree = 5, Clustering Coefficient = 0.60, Radiality = 3.64), adenosine deaminase (*ADA*;MCC = 144,Degree = 6, Clustering Coefficient = 0.87, Radiality = 3.64), adenylate kinase 2 (*AK2*;MCC = 288,Degree = 8, Clustering Coefficient = 0.71, Radiality = 3.86), adenylate kinase 7 (*AK7*;MCC = 296,Degree = 9, Clustering Coefficient = 0.64, Radiality = 3.93), adenosine kinase (*ADK*;MCC = 292,Degree = 10, Clustering Coefficient = 0.49, Radiality = 4.07), ectonucleoside triphosphate Di phosphohydrolase 2(*ENTPD2*;MCC = 48,Degree = 5, Clustering Coefficient = 0.90, Radiality = 3.57), and nucleoside diphosphate kinase A(*NME1*;MCC = 29, Degree = 7, Clustering Coefficient = 0.38, Radiality = 3.79) were detected among the PPI network based on MCC analysis (Fig. 6A). After overlap, the hub genes, includes *IMPDH1*, *ENTPD2*, *AK7*, *AK2*, and *CANT1*, were identified in Fig. 6B. The heatmap of expression profile showed that there were significant differences in the expression level (Fig. 6C). This plot of Fig. 6D depicts the how hub gene’s expression levels were in relation to SHAP values. Consequently, according to the model, the lower the SHAP value, corresponding to the higher expression level of these genes, may suggesting the more likely DCM becomes.

### Validation of purine metabolism related hub pathway and gene

**Fig. 7:**
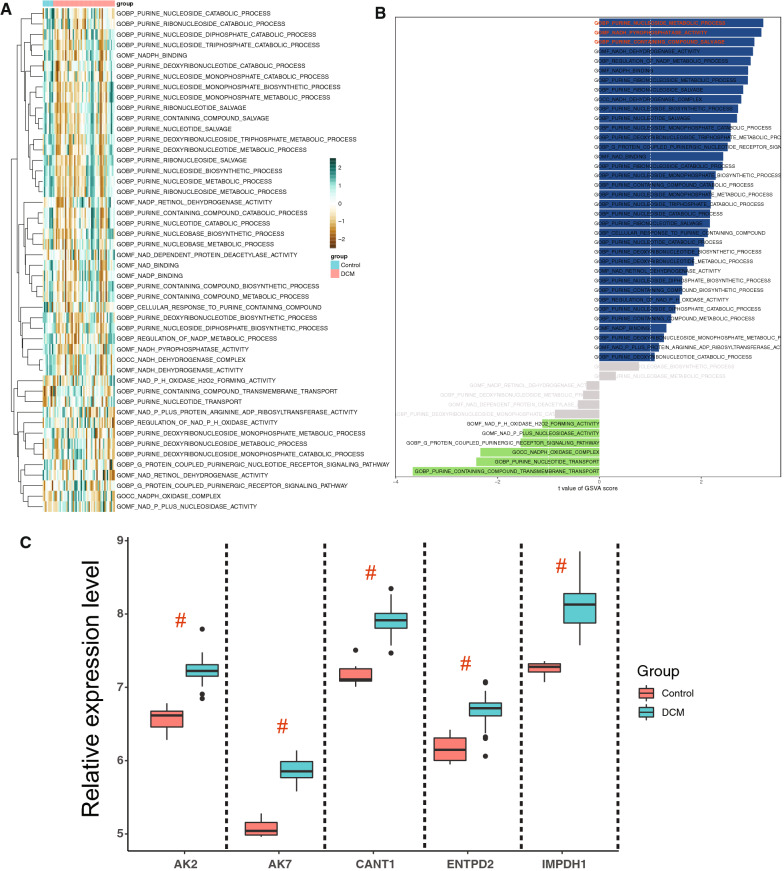
Validation of hub gene expression and purine metabolism pathway. **A**, **B** The heatmap and bar plot showing the GSVA score of the purine metabolism related pathway, and difference analysis among DCM and control samples. **C** After normalized, the expression levels of *IMPDH1*, *ENTPD2*, *AK7*, *AK2*, and *CANT1* were verified in GSE120895 dataset. All of the markers were significantly higher in DCM left ventricular samples than the control groups

Additionally, hub genes were validated using the external dataset of GSE120895. Consequently, the results shown that the bulk of purine metabolism related pathway presenting a significant difference between the control and DCM groups (Fig. 7A, B). After RMA-normalized, the relative expression level of *IMPDH1*, *ENTPD2*, *AK7*, *AK2*, and *CANT1* were significantly higher in DCM left ventricular samples than the control groups (Fig. 7C).

## Discussion

Based on the integrated analysis of transcriptomics and complete-spectrum metabolomics, we found that purine metabolism may be a prominent metabolic change in cardiac pathological progression of patients with DCM. Purine metabolism is involved in the normal energy flow of the heart. Purines are often distributed in DNAs and RNAs, catalyzed by various oxidases to form hypoxanthine and xanthine, then oxidized by urate oxidase to realize uric acid metabolism. Evidence is accumulating that levels of purine degradation intermediates indicate the energy state of myocardial cells. In physiological conditions, if energy consumption of the heart increases, purine nucleotides and their metabolites also increase; conversely, a distinctive reduction in total purine release suggests that myocardial cells relatively conserve energy and maintain the energy state of the myocardium. In pathological conditions, when myocardial ischemia occurs, ATP is degraded into xanthine and accumulated in tissues, and massive xanthine in myocardial cells is then degraded into uric acid by xanthine oxidase, with simultaneous production of superoxide anions in a large number, causing pathological damage of the cells. In hypoxic conditions, the degradation products of adenosine and inosine are better energy sources than extracellular glucose, which further delay the accumulation of nicotinamide adenine dinucleotide (NADH) and display a certain protective effect on the cells. DCM patients with heart failure have abnormal energy metabolism, and this condition can further aggravate damage of myocardial cell structure and function, namely, “myocardial metabolic remodeling”, with the two factors mutually influencing each other.

The *IMPDH1* encoded the rate-limiting enzyme in the de novo synthesis of xanthine monophosphate (XMP) from inosine-5'-monophosphate (IMP). As reported, *IMPDH1* function as the catalyzes in the development of multiple organs and progression of cardiovascular disease. Kofler et al., found that the inosine monophosphate dehydrogenase (*IMPDH*) activity may significantly correlated with the incidence of acute rejection episodes and transplant vasculopathy based on a prospective study [41]. Ohmann et al., demonstrated that the haplotype of *IMPDH1*, includes the SNPs of rs2288553, rs2288549, rs2278293, rs2278294, and rs2228075, may strongly associated with the gastrointestinal related side effects of immunosuppressive therapy after heart transplantation in pediatric patients [42].Burckart and Amur reviewed that the polymorphisms of *IMPDH1* and *IMPDH2* were detected which’re significantly correlated with the static graft, survival rate, and the incidence of adverse drug effects in heart transplant patients [43]. The *ENTPD2* is encoded type 2 enzyme of the family of ECTO-nucleoside triphosphate diphosphohydrolase (E-NTPDase), playing an important role in hydrolyze 5'-triphosphates and maintain the stability of cell membrane protein. During the normal systolic and diastolic physiological activities of mice, Rücker et al., via detects the functional state of mitochondria, pH value, ATP and ADP hydrolysis in heart tissue, and thus found that the cation-dependent enzymes of ATP and ADP hydrolysis optimum pH is 8.0, and AMP hydrolysis is 9.5. Additionally, the content of *ENTPD2* gene and protein was the highest expression in the left ventricle of mice [44]. It is suggested that a play an important role in maintaining normal cardiac diastolic and systolic function. Bertoni et al., also found that the activity of ENTPD2 may play a significant role in regulate the extracellular ATP and adenosine levels during the pathological process of vascular smooth muscle cell plasticity [45]. *CANT1* is a subset of cell growth factor of apyrase family and functions as a calcium-dependent nucleotidase with a preference for urinary dihydrate (UDP); it plays a key role in the pathophysiological process involved in calcium ion binding and pyrophosphatase activity. Yang et al., found that the *CANT1* expression level is closely related with TP53-mutantation and poor prognosis of hepatocellular carcinoma [46]. Based on whole-exome sequencing (WES) analysis of three patients from two unrelated families, Byrne et al. found that a variant of *CANT1* may contribute to the pathogenesis of pseudodiastrophic dysplasia related cardiac developmental defect [47]. Jelin et al., also reported that the mutations in *CANT1* may significantly correlated with specific skeletal dysplasias in the neonatal dysplasia [48]. *AK2* and *AK9* are belong to the family proteins of adenylate kinases, that catalyze the production and breakdown of adenine nucleotide composition with the reversible transfer method. However, *AK2* and AK9 shown a tissue-specific and developmentally regulated in different physiological and pathological processes. Isozyme-2 of adenylate kinase is major localized in the mitochondrial intermembrane space and presenting an important role in regulation of catalase, oxidase cell apoptosis. Zhang et al., illustrated that the *AK2* deletion wound lead to fetal intrauterine death. And in adult mice, organ-specific ablation of *AK2* can lead to heart failure, which may be related to metabolic dysfunction involved in Krebs cycle and glycolytic metabolite buildup [49]. As the key metabolic sensor of cell energy economy, the expression level of *AK2* play an important role in regulate metabolic signaling circuits, nuclear transport, and energetics of cell cycle involving in DNA synthesis and repair [50]. Carrasco et al., found that the deletion of *AK2* may compromise the nucleotide exchange in the mitochondrial intermembrane space, and thus regulate the balance of mitochondria energetics with K(ATP) channels [51]. The enzyme Adenylate Kinase 7 (*AK7*) may function as the phosphotransferase, and plays a role in energy homeostasis. Romeo-Guitart et al., found that *AK7* may significantly correlated with intracellular endoplasmic reticulum (ER) stress and the activation of unfolded protein response [52]. Lorès et al., shown that the homozygous missense mutation L673P leads to the deletion of Ak7 protein, and thus result in the injury of mitochondria respiratory function and dysfunction of assembly [53].

The function of stem cell in the heart can be enhanced by biomaterials [54], and it has good therapeutic effect on heart failure. Heart failure is caused by primary myocardial cell dysfunction (such as hereditary cardiomyopathy) or myocardial cell loss (such as after myocardial infarction). The current drug treatment has reduced the mortality and morbidity, but can not produce new myocardial cells [55]. The natural materials commonly used in heart tissue engineering include collagen, elastin, gelatin, fibrin, chitosan and silk fibroin [56], and they have proper biochemical characteristics of cell attachment and proliferation [57]. Engineered heart tissue (EHT) based on novel biomaterials or nanomaterials is a promising method to treat heart failure. It is mainly to obtain mature EHT in drug screening or cell therapy, use natural biological materials or synthetic nanomaterials to provide mechanical support, and generate 2D or 3D myocardial cell slices with non-shrinking cells [58]. Nevertheless, researchers are faced with many obstacles in translating the application of biomaterials into clinical practice [59]. Finding an ideal biomaterial is still challenging. It should be close to the natural extracellular matrix cells to survive, strengthen the coupling between donor and host cells, and have no immune response after degradation. Using stem cells and bioengineering technology to develop EHT technology will provide mature myocardial cells to supplement lost myocardium, repair scar tissue, and bear local mechanical and hemodynamic loads imposed on them. In addition to manipulating EHT in vitro, researchers can also optimize the host substrate environment by targeting fibroblast activation pathways or modifying ECM to promote cell implantation and functional integration of newborn cardiomyocytes. Good biomaterials can be combined in stem cell therapy. These intervention methods need further research, combined with EHT technology, to ensure the efficacy of heart failure treatment.

Studies have shown that ventricular remodeling and myocardial energy metabolism remodeling are the basis of heart failure [60]. Mitochondria are the main energy source of myocardial cells, accounting for about one-third of the volume of myocardial cells, and myocardial cells also have high metabolic activity [61]. Therefore, new cardiovascular diseases can be developed for mitochondria. Heart failure is closely related to energy deficiency and mitochondrial dysfunction [62]. The mitochondrial dysfunction of heart failure may provide a new method, which is not only conducive to hemodynamics, but also a supplement to the existing limited methods. However, up to now, mitochondrial targeted therapy has not successfully affected the progress of this disease [63]. Compared with other organs, the heart needs a lot of energy. About a third of an adult cardiomyocyte is made up of mitochondria. Most of the energy consumed by the heart is provided by oxidative metabolism of mitochondria, and the key mechanism of cardiac systolic failure is the inability to produce and transfer energy. However, more and more people are realizing that mitochondria not only provide energy, but also play important biological and regulatory roles, such as cell growth and death, protein quality control, REDOX balance, ion homeostasis, biosynthesis, reactive oxygen species (Ros) signaling, etc. Researchers are beginning to realize that the pathogenic role of mitochondria in cardiovascular disease and heart failure is not only related to decreased ATP production, but also to general maladaptation of the functional spectrum [64]. But the integration of mitochondrial bioenergetics into each behavior is poorly understood, and the contribution of each unique biological function of mitochondria to the development of heart failure remains unclear. In addition. Further elucidation of the linkages between the many other functions of mitochondria and the processes involved in oxidative metabolism may help to discover new therapeutic targets. The renin-angiotensin system (RAS) is involved in cardiovascular disease risk factors and is an enzymatic pathway that promotes cardiovascular disease (CVD) and the progression of cardiovascular disease. Renin mediates the conversion of angiotensinogen to the inactive polypeptide angiotensin I and then to the active hormone angiotensin II (Ang II) [65]. As a pro-oxidant and fibroblast factor, angiotensin II (Ang II) is the main effector peptide of RAS [66]. The increase of angiotensin ii and superoxide disproportionation in central and peripheral nervous system play a role in enhancing sympathetic vasomotor tension in heart failure. RAS activity and oxidative stress are gradually increased during the development of heart failure [67]. Drugs that target various components of the systemic RAS, including angiotensin ii type 1 receptor blockers (ARBs), renin inhibitors, and angiotensin converting enzyme inhibitors, are used to treat heart failure [68].

In this study, due to the complexity of collecting samples, not many samples were included. New samples should be collected in future work to further verify our findings. In addition, we should further verify the expression level of AK2, AK7, CANT1, ENTPD2, IMPHD1 and other marker genes in DCM patients, and screen important pathways for further in-depth research. In addition, in future research, we will collect external data to further verify our results, and conduct internal cross validation to further ensure the accuracy of our conclusions.

## Conclusions

The present study demonstrated a significant upregulation in the expression levels of two metabolites, guanine and urea, accompanied by a significant downregulation of xanthine, in myocardial tissues of DCM. However, the major regulatory genes that cause changes in the above-mentioned metabolites and DCM phenotype may be *IMPDH1*, *ENTPD2*, *AK7*, *AK2*, and *CANT1*. These differential genes and metabolites are likely to serve as vital markers for diagnosis or treatment during heart failure progression in DCM. In the future work, it is suggested to take IMPDH1、ENTPD2、AK7、AK2 and CANT1 as targets to develop drugs for the treatment of chronic HF, and Improve the physical condition of HF patients by regulating purine metabolism.

## Supplementary Information

**Additional file 1: Table S1.** The gene list of the differential expressed genes analysis in GSE57338 dataset.

**Additional file 2: Table S2.** The list of pathway analysis results following the gene-set enrichment analysis.

**Additional file 3: Table S3.** The differential analysis results of metabolism pathway following the gene set variation analysis.

**Additional file 4: Table S4.** The results of metabolite expression profiles and differentially expressed metabolites analysis.

**Additional file 5: Table S5.** The differential analysis results of purine metabolism related pathway basing on the gene set variation analysis.

## Data Availability

The datasets of this article are available in the Gene Expression Omnibus database (GEO; https://www.ncbi.nlm.nih.gov/geo/; GSE57338, GSE19303, and GSE120895). The metabolic profiling data and clinical features can be accessed from corresponding author or first author upon request.
